# Asymptotic analysis of particle cluster formation in the presence of anchoring sites

**DOI:** 10.1140/epje/s10189-024-00425-8

**Published:** 2024-05-08

**Authors:** Paul C. Bressloff

**Affiliations:** Department of Mathematics, Imperial College London, London, SW7 2AZ UK

## Abstract

**Abstract:**

The aggregation or clustering of proteins and other macromolecules plays an important role in the formation of large-scale molecular assemblies within cell membranes. Examples of such assemblies include lipid rafts, and postsynaptic domains (PSDs) at excitatory and inhibitory synapses in neurons. PSDs are rich in scaffolding proteins that can transiently trap transmembrane neurotransmitter receptors, thus localizing them at specific spatial positions. Hence, PSDs play a key role in determining the strength of synaptic connections and their regulation during learning and memory. Recently, a two-dimensional (2D) diffusion-mediated aggregation model of PSD formation has been developed in which the spatial locations of the clusters are determined by a set of fixed anchoring sites. The system is kept out of equilibrium by the recycling of particles between the cell membrane and interior. This results in a stationary distribution consisting of multiple clusters, whose average size can be determined using an effective mean-field description of the particle concentration around each anchored cluster. In this paper, we derive corrections to the mean-field approximation by applying the theory of diffusion in singularly perturbed domains. The latter is a powerful analytical method for solving two-dimensional (2D) and three-dimensional (3D) diffusion problems in domains where small holes or perforations have been removed from the interior. Applications range from modeling intracellular diffusion, where interior holes could represent subcellular structures such as organelles or biological condensates, to tracking the spread of chemical pollutants or heat from localized sources. In this paper, we take the bounded domain to be the cell membrane and the holes to represent anchored clusters. The analysis proceeds by partitioning the membrane into a set of inner regions around each cluster, and an outer region where mean-field interactions occur. Asymptotically matching the inner and outer stationary solutions generates an asymptotic expansion of the particle concentration, which includes higher-order corrections to mean-field theory that depend on the positions of the clusters and the boundary of the domain. Motivated by a recent study of light-activated protein oligomerization in cells, we also develop the analogous theory for cluster formation in a three-dimensional (3D) domain. The details of the asymptotic analysis differ from the 2D case due to the contrasting singularity structure of 2D and 3D Green’s functions.

**Graphical abstract:**

2D model of diffusion-based protein cluster formation in the presence of anchoring cites and particle recycling. **a** A set of *N* anchoring sites at positions ${\textbf{x}}_j$, $j=1,\ldots ,N$, in a bounded domain $\Omega$. **b** Diffusing particles accumulate at the anchoring sites resulting in the formation of particle aggregates or clusters ${{\mathcal {U}}}_j$. **c** The clusters are dynamically maintained by a combination of lateral diffusion outside the clusters and particle recycling
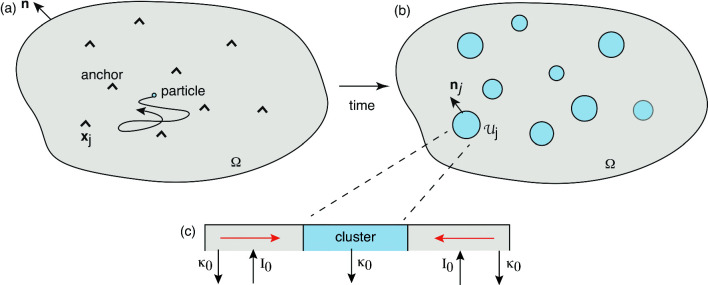

## Introduction

The aggregation or clustering of proteins and other macromolecules plays an important role in the formation of large-scale molecular assemblies in cells. In many cases, such assemblies are associated with cellular membranes, including the clustering of cell-cell adhesion proteins in epithelia [1] and lipid raft formation [2, 3]. Another notable example is the formation of postsynaptic domains (PSDs) at excitatory and inhibitory synapses in neurons. PSDs are rich in scaffolding proteins that can transiently trap transmembrane neurotransmitter receptors, thus localizing them at specific spatial positions, in particular, at sites apposed to active zones in presynaptic domains where neurotransmitters are released. PSDs thus play a crucial role in determining the effective strength of synaptic connections between cells [4–12]. Advances in single-particle tracking and imaging methods have shown that PSDs are highly dynamic structures whose constituent molecular components are subject to continuous turnover. For example, scaffolding protein-receptor complexes can diffuse laterally within the cell membrane. A surface complex may also be internalized via endocytosis and stored within an intracellular compartment, where it is either recycled to the surface via recycling endosomes and exocytosis, or sorted for degradation by late endosomes and lysosomes.

A number of models have explored the combined effects of diffusion-trapping and recycling on the number of excitatory AMPA ($\alpha$-amino-3-hydroxy-5-methyl-4-isoxazolepropionic) receptors within dendritic spines [13–18]. However, these diffusion-trapping models typically assume that the number of trapping sites or “slots” within a given PSD is fixed. In order to understand the formation and stabilization of PSDs, it is necessary to consider the slower dynamics of scaffolding protein-receptor complexes. Several modeling studies have analyzed the joint localization of gephyrin scaffolding proteins and glycine receptors at inhibitory synapses [19–21], showing how stable PSDs could arise dynamically via a non-equilibrium Turing mechanism. An alternative approach is to consider PSD formation in terms of diffusion-mediated particle aggregation or coalescence within the cell membrane [22, 23]. The fusion of smaller diffusing clusters and particles is modeled according to Smoluchowski coagulation equations, and the system is kept out of equilibrium by the recycling of particles between the cell membrane and interior [24]. This allows for the formation and maintenance of a stationary distribution consisting of multiple clusters. Recently, the PSD aggregation model has been extended to include fixed anchoring sites that trap clusters at specific positions within the membrane, consistent with the alignment of PSDs and presynaptic active zone [25]. The effects of the anchoring sites were analyzed using a mean field description of the steady-state particle concentration around a single cluster. This yielded an expression for the mean cluster size as a function of model parameters such as the density of anchoring sites.

As far as we are aware, current mathematical/computational models of clustering have not been compared directly with detailed experimental studies of PSD formation. However, they do incorporate the various molecular players and biophysical mechanisms that have been identified experimentally as playing a crucial role in PSD formation. Modeling studies thus establish a proof of principle for the hypothesized processes and provide information regarding expected cluster sizes and distributions [22, 23, 25]. There is an analogous role for modeling in studies of the role of liquid-liquid phase separation in the formation of biological condensates. Many different types of biological condensate are found intracellularly in the cytoplasm and cell nucleus (see the reviews [26–29] and references therein). These membrane-less organelles are viscous, liquid-like structures containing enhanced concentrations of various proteins and RNA, many of which can be continually exchanged with the surrounding medium.

It has been hypothesized that the coexistence of multiple droplets over significant timescales involves the active suppression of Ostwald ripening. Ostwald ripening describes the coarsening of droplets during late-stage liquid-liquid phase separation via spinodal decomposition [30, 31] and is distinct from the coarsening process underlying aggregation models. In classical Ostwald ripening, an emulsion of polydisperse droplets transitions to a single condensate in thermodynamic equilibrium with a surrounding dilute phase. Various hypotheses have been given to account for the suppression of Ostwald ripening including the following: actively driven chemical reactions that maintain the out-of-equilibrium switching of proteins between soluble and phase separating forms [32–35]; the mechanical suppression of coarsening mediated by intracellular visco-elastic networks such as the cytoskeleton [36–38]; the slow conversion of a molecular constituent between the dilute and dense phases due to various regulatory interfacial proteins [39–41]. As in the case of models of clustering, mean field approximations are often used to analyze the effects of diffusion on the formation and maintenance of multiple droplets.

Recently, we have extended mean field models of Ostwald ripening using the theory of diffusion in singularly perturbed domains [42–44]. The latter is a powerful analytical method for solving boundary value problems (BVPs) for two-dimensional (2D) and three-dimensional (3D) diffusion in domains where small holes or perforations have been removed from the interior [45–54]. Applications range from modeling intracellular diffusion, where interior holes could represent subcellular structures such as organelles, molecular clusters and liquid droplets, to tracking the spread of chemical pollutants or heat from localized sources. Roughly speaking, one can divide the various BVPs into two distinct groups. The first, which is relevant to the current paper, treats the holes as localized sources or reflecting obstacles for populations of particles, and one is interested in calculating the steady-state solution (if it exists) and the rate of approach to steady state. The second treats the holes as totally or partially absorbing traps, and the main focus is determining the first passage time or splitting probability for a single particle to be captured by an interior trap (narrow capture).

Both types of BVP can be solved using a combination of matched asymptotic analysis and Green’s function methods. This involves obtaining an inner or local solution of the diffusion equation that is valid in a small neighborhood of each hole, and then matching to an outer or global solution that is valid away from each neighborhood. The matching requires taking into account the singular nature of the Green’s function associated with the diffusion equation. However, the details of the matched asymptotic analysis in 2D and 3D domains differ considerably due to corresponding differences in the Green’s function singularities. That is, as $|{\textbf{x}}-{\textbf{x}}_0|\rightarrow 0$,

$$\begin{aligned}&G({\textbf{x}},{\textbf{x}}_0)\rightarrow -\frac{1}{2\pi D}\ln |{\textbf{x}}-{\textbf{x}}_0| \text{ in } \text{2D } \\&G({\textbf{x}},{\textbf{x}}_0)\rightarrow \frac{1}{4\pi D|{\textbf{x}}-{\textbf{x}}_0|} \text{ in } \text{3D }. \end{aligned}$$

Consequently, an asymptotic expansion of the solution to a BVP in 3D is in powers of $\epsilon$, where $\epsilon$ represents the size of a hole relative to the size of the bulk domain. On the other hand, the analogous expansion in 2D tends to be in powers of $\nu =-1/\ln \epsilon$ at *O*(1) in $\epsilon$. The slower convergence of $\nu$ in the limit $\epsilon \rightarrow 0$ can be dealt with by summing the logarithmic terms non-perturbatively [45, 46]. Note that the only major constraints on the applicability of the method is that the holes are much smaller than the size of the domain, and are well separated from each other and the boundary of the domain. The latter two constraints can be relaxed, but the analysis is significantly more difficult.

In this paper, analogous to our previous work on Ostwald ripening [42–44], we use the theory of diffusion in singularly perturbed domains to extend the mean field analysis of cluster formation in the presence of anchoring sites. This allows us to take into account diffusion-mediated interactions between the anchored clusters. In order to highlight the generality of the methods used in this paper, we also briefly recap the analysis of classical Ostwald ripening. The structure of the paper is as follows. In Sect. 2, we begin by presenting the 2D aggregation model introduced in Ref. [25], together with its mean field formulation. We then consider a 3D version of the model. One major difference from the 2D model is that the recycling of particles can no longer be interpreted in terms of membrane exo/endocytosis. Instead, we assume that particles exist in either an active or inactive state, and can only form clusters in the active state. One motivation for the 3D model is a recent study of the optogenetic protein CRY2olig, which oligomerizes (forms small clusters) in the presence of blue light [55]. (In this particular study, the authors explore both theoretically and experimentally the effects of obstacles on the formation of large 3D protein clusters via the diffusion-limited aggregation of oligomers.) We end Sect. 2 by briefly reviewing the corresponding mean field treatment of classical Ostwald ripening.

Since the theory of diffusion in singularly perturbed domains is probably unfamiliar to the broad statistical physics community, we provide a detailed overview of the method in Sect. 3 by considering a simplified problem in which the holes are of a known fixed size. We highlight the differences between 2D and 3D diffusion due to the different singularity structure of the corresponding Green’s functions. As a point of comparison with the clustering model, we briefly indicate how the asymptotic analysis can be used to determine corrections to the mean field kinetics of droplets during Ostwald ripening. We then apply the theory in Sects. 4 and 5 to the 2D and 3D clustering models, respectively. In particular, we derive expressions for the steady-state particle concentration outside the clusters that depend on the positions of the various anchor points and the boundary of the domain. The steady-state cluster sizes are then obtained by calculating the total flux of particles at the surface of each cluster.

## Cluster formation model and the mean field approximation

In this section, we summarize the 2D diffusion model of clustering and its mean field approximation, which was formulated in Ref. [25]. We then show how to extend the mean field theory to a 3D clustering model, and briefly describe classical Ostwald ripening.

### Diffusion model of clustering in 2D

**Fig. 1:**
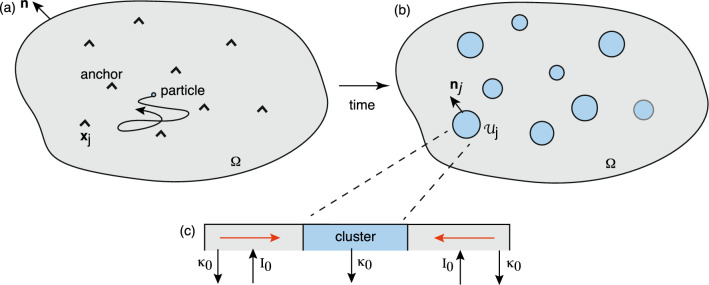
2D model of diffusion-based protein cluster formation in the presence of anchoring cites and particle recycling. **a** A set of *N* anchoring sites at positions ${\textbf{x}}_j$, $j=1,\ldots ,N$, in a bounded domain $\Omega$. **b** Diffusing particles accumulate at the anchoring sites resulting in the formation of particle aggregates or clusters ${{\mathcal {U}}}_j$. **c** The clusters are dynamically maintained by a combination of lateral diffusion outside the clusters and particle recycling

Consider a 2D bounded domain $\Omega \subset {{\mathbb {R}}}^2$ containing a set of *N* anchoring sites ${\textbf{x}}_j \in \Omega$, $j=1,\ldots ,N$, see Fig. 1a. Suppose that at time *t*, $t\ge 0$, there exists a circularly symmetric cluster ${{\mathcal {U}}}_j(t)$ of radius $R_j(t)$ at the *j*th site, see Fig. 1b. That is,

2.1 $$\begin{aligned} {{\mathcal {U}}}_j(t)=\{{\textbf{x}}\in \Omega ,\ |{\textbf{x}}-{\textbf{x}}_j|\le R_j(t)\}. \end{aligned}$$

Let $c({\textbf{x}},t)$, ${\textbf{x}}\in \Omega$, denote the concentration of freely diffusing particles (monomers), which evolves according to the diffusion equation

2.2a $$\begin{aligned} \frac{\partial c({\textbf{x}},t)}{\partial t}&= D{\varvec{\nabla }}^2 c({\textbf{x}},t)-\kappa _0 c({\textbf{x}},t)+I_0,\nonumber \\&{\textbf{x}}\in \Omega \backslash \cup _{j=1}^N {{\mathcal {U}}}_j(t), \end{aligned}$$

2.2b $$\begin{aligned} D{\varvec{\nabla }} c({\textbf{x}},t) \cdot {\textbf{n}}&=0,\ {\textbf{x}}\in \partial \Omega ,\end{aligned}$$

2.2c $$\begin{aligned} c({\textbf{x}},t)&=0,\quad {\textbf{x}}\in \partial {{\mathcal {U}}}_j(t). \end{aligned}$$

Here ${\textbf{n}}$ is the outward unit normal to the surface $\partial \Omega$, the constant $\kappa _0$ denotes the recycling or turnover rate of individual particles outside a cluster, and $I_0$ is the re-injection flux of recycled particles, see Fig. 1c. We take the exterior boundary $\partial \Omega$ to be totally reflecting so that the total number of particles (freely diffusing and clustered) is conserved. Also note that the set notation $A\backslash B$ means the domain *A* excluding *B*, that is, $A\backslash B=A \cap B^c$ where $B^c$ is the complement of *B*. In particular, $\Omega \backslash \cup _{i=1}^N{{\mathcal {U}}}_{i},$ is the domain exterior to the clusters.)

It remains to specify the dynamics of cluster growth. Let ${{\mathcal {N}}}_j(t)$ denote the total number of particles within the *j*th cluster at time *t*. It follows from particle conservation that

2.3 $$\begin{aligned} \frac{d}{\text {d}t}{{\mathcal {N}}}_j(t)&= D\int _{\partial {{\mathcal {U}}}_j(t)}{\varvec{\nabla }} c({\textbf{x}},t) \cdot {\textbf{n}}_jd{\textbf{x}}-\kappa _0 {{\mathcal {N}}}_j(t), \end{aligned}$$

where $\textbf{n}_j$ is the outward unit normal to the surface $\partial {{\mathcal {U}}}_j$. In the case of a uniform density $u_0$ of particles within a cluster, the number of particles is ${{\mathcal {N}}}_j(t)=|{{\mathcal {U}}}_j(t)|u_0$. For concreteness, we assume that the rate of recycling within a cluster is also $\kappa _0$, and that no particles are re-injected directly into a cluster (as appears to hold for PSD formation). However, one could consider a more general model, in which the recycling rates within and outside clusters differ.

In this paper, we are interested in calculating the steady-state solution. We proceed by solving the steady-state version of Eq. (2.2a–c) in terms of the unknown steady-state radii $R_j$, $j=1,\ldots ,N$:

2.4a $$\begin{aligned} D{\varvec{\nabla }}^2 c({\textbf{x}})-\kappa _0 c({\textbf{x}})+I_0&=0,\ {\textbf{x}}\in \Omega \backslash \cup _{j=1}^N {{\mathcal {U}}}_j, \end{aligned}$$

2.4b $$\begin{aligned} D{\varvec{\nabla }} c({\textbf{x}}) \cdot {\textbf{n}}&=0,\ {\textbf{x}}\in \partial \Omega ,\end{aligned}$$

2.4c $$\begin{aligned} c({\textbf{x}})&=0,\quad {\textbf{x}}\in \partial {{\mathcal {U}}}_j. \end{aligned}$$

The radii are then determined self-consistently by imposing the steady-state version of Eq. (2.2d):

2.5 $$\begin{aligned} J_j:=D\int _{\partial {{\mathcal {U}}}_j}{\varvec{\nabla }} c({\textbf{x}}) \cdot {\textbf{n}}_jd{\textbf{x}}=\kappa _0 u_0 |{{\mathcal {U}}}_j| \end{aligned}$$

for $j=1,\ldots ,N$, where $J_j$ is the total flux into the cluster.

For the moment, suppose that each cluster is treated as a point source/sink. That is, we replace Eq. (2.4) by

2.6 $$\begin{aligned} D{\varvec{\nabla }}^2 c({\textbf{x}})-\kappa _0 c({\textbf{x}})+I_0-\sum _{j=1}^N J_j \delta ({\textbf{x}}-{\textbf{x}}_j)&=0, \end{aligned}$$

together with the boundary conditions $c({\textbf{x}}_j)=0$, $j=1,\ldots ,N$. Consider the Neumann Green’s function $G({\textbf{x}},{\textbf{y}})$ for the modified Helmholtz equation, which is uniquely defined by

2.7a $$\begin{aligned} D{\varvec{\nabla }}^2 G-\kappa _0 G&=-\delta ({\textbf{x}}-{\textbf{x}}'),\quad {\textbf{x}},{\textbf{x}}' \in \Omega , \end{aligned}$$

2.7b $$\begin{aligned} {\varvec{\nabla }G}\cdot {\textbf{n}}&=0 \text{ on } \partial \Omega \end{aligned}$$

for fixed ${\textbf{x}}'$. Note that in 2D *G* can be decomposed as

2.8 $$\begin{aligned} G({\textbf{x}},{\textbf{x}}')=-\frac{ \ln |{\textbf{x}}-{\textbf{x}}'|}{2\pi D}+{{\mathcal {R}}}({\textbf{x}},{\textbf{x}}'), \end{aligned}$$

where ${{\mathcal {R}}}$ is the regular (non-singular) part of the Green’s function. It follows that the formal solution of Eq. (2.6) is given by

2.9 $$\begin{aligned} c({\textbf{x}})&=\int _{\Omega } G({\textbf{x}},{\textbf{x}}')\left[ I_0-\sum _{k=1}^NJ_j \delta ({\textbf{x}}'-{\textbf{x}}_k)\right] d{\textbf{x}}'\nonumber \\&=c_0-\sum _{k=1}^NJ_kG({\textbf{x}},{\textbf{x}}_k), \end{aligned}$$

where $c_0=I_0/\kappa _0$ and we have used the fact that $\int _{\Omega }G({\textbf{x}},{\textbf{x}}')d{\textbf{x}}'=\kappa _0^{-1}$.

In principle, the unknown fluxes $J_j$ could now be obtained by imposing the conditions $c({\textbf{x}}_j)=0$, which would yield the matrix equation $c_0=\sum _{k=1}^NG({\textbf{x}}_j,{\textbf{x}}_k)J_k$ for $j=1,\ldots ,N$. However, the Green’s function $G({\textbf{x}},{\textbf{x}}')$ has a logarithmic singularity in the limit ${\textbf{x}}\rightarrow {\textbf{x}}'$, so that the diagonal elements $G({\textbf{x}}_j,{\textbf{x}}_j)=\infty$. We conclude that in 2D one cannot treat the clusters as point-like objects. (An analogous result holds for 3D diffusion due to the $1/|{\textbf{x}}-{\textbf{x}}'|$ singularity of the 3D Green’s function.) This issue was dealt with in Ref. [25] by considering a mean field approximation as detailed below. One of the main goals of this paper is to show how one can analyze the full steady-state Eq. (2.4) using asymptotic methods that provide a systematic procedure for handling the Green’s function singularities.

### Mean field approximation

The mean field approximation of Ref. [25] involves coarse-graining the system by treating each cluster as if it is in a “sea” of uniformly distributed background clusters. Suppose that all of the clusters have the same radius *R* and that the background cluster density is $\phi _0$. Ignoring the effects of the boundary $\partial \Omega$, we can take the concentration *c* around a single cluster to be circularly symmetric. The coarse-grained version of Eq. (2.6) for any individual cluster is then

2.10 $$\begin{aligned} D{\varvec{\nabla }}^2 c(r)-\kappa _0 c(r)+I_0-\phi _0 J(R)&=0,\ r >R, \end{aligned}$$

with $c(R)=0$ and the flux *J*(*R*) into the surface of each cluster determined self-consistently from the equation

2.11 $$\begin{aligned} J(R)=2\pi R D\partial _rc(R). \end{aligned}$$

This 2D mean-field approximation was analyzed in Ref. [25]. First, the solution of Eq. (2.10) is given by

2.12 $$\begin{aligned} c(r)=\left( c_0-\frac{\phi _0J(R)}{\kappa _0}\right) \left( 1-\frac{K_0(r/\lambda )}{K_0(R/\lambda )}\right) , \end{aligned}$$

where $K_{\nu }$ is a modified Bessel function of the second kind, $c_0=I_0/\kappa _0$, and $\lambda =\sqrt{D/\kappa _0}$. Substituting the solution into Eq. (2.11) yields a self-consistency equation for the flux *J*(*R*):

2.13 $$\begin{aligned} J(R)=\frac{2\pi RD}{\lambda } \left( c_0-\frac{\phi _0J(R)}{\kappa _0}\right) \frac{K_1(R/\lambda )}{K_0(R/\lambda )}. \end{aligned}$$

We have used the Bessel identity $K_0'(x)=-K_1(x)$. Equation (2.13) can be rewritten as an implicit equation for the cluster radius *R* by using Eq. (2.5), since $J(R)=\pi R^2 \kappa _0 u_0$ [25]:

2.14 $$\begin{aligned} \frac{R}{\lambda }=2 \left( \frac{c_0}{u_0}-\pi \left( \frac{R}{\lambda }\right) ^2 \phi _0\lambda ^2\right) \frac{K_1(R/\lambda )}{K_0(R/\lambda )}. \end{aligned}$$

The mean cluster radius *R* can now be determined as a function of model parameters by solving Eq. (2.14) numerically [25]. In anticipation of the subsequent asymptotic analysis, suppose $\phi _0 \pi \lambda ^2 \ll 1$ (dilute cluster regime), so that

2.15 $$\begin{aligned} \frac{R}{\lambda }\approx \frac{2c_0}{u_0}f(R/\lambda ),\quad f(R/\lambda )=\frac{K_1(R/\lambda )}{K_0(R/\lambda )}. \end{aligned}$$

**Fig. 2:**
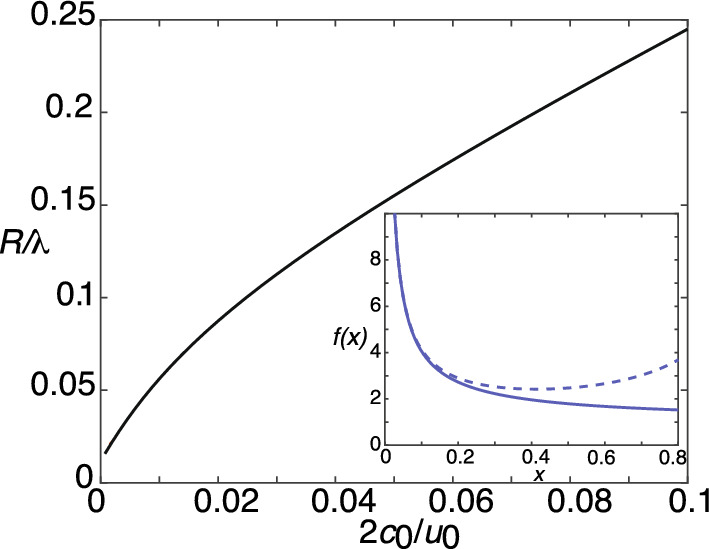
Mean-field approximation in the dilute cluster regime. Plot of non-dimensionalized mean cluster radius $R/\lambda$ as a function of $2c_0/u_0$, where $c_0$ is the stationary particle concentration away from clusters and $u_0$ is the particle concentration within a cluster. *Inset*: Plot of $f(x)=K_1(x)/K_0(x)$ as a function of *x* (solid curve). The asymptotic approximation of *f*(*x*) obtained from Eq. (2.16) is shown as the dashed curve

The solution for $R/\lambda$ is plotted as a function of the parameter $2c_0/u_0$ in Fig. 2. It can be seen that if $2c_0/u_0$ is sufficiently small $\Gamma$ (as assumed in Ref. [25]), then the mean cluster radius is relatively small. Therefore, we can exploit the small-*z* expansions (see inset of Fig. 2)

2.16 $$\begin{aligned} K_0(z)\sim -\ln (z/2) -\gamma _c,\quad K_1(z)\sim \frac{1}{z}, \end{aligned}$$

where $\gamma _c \approx 0.5772$ is Euler’s gamma constant. Setting $\epsilon ^2=2c_0/u_0$ and $R/\lambda =\epsilon \rho /\lambda$ with $\rho /\lambda =O(1)$, we thus find that

2.17 $$\begin{aligned} \left( \frac{ \rho }{\lambda }\right) ^2&\sim \frac{1}{-\ln \epsilon +\ln 2-\ln \rho /\lambda -\gamma _c}\nonumber \\&= \frac{\nu }{1+\nu \left( \ln 2-\ln \rho /\lambda - \gamma _c\right) }, \end{aligned}$$

where $\nu =-1/\ln \epsilon$. As we highlighted in the introduction, the non-perturbative dependence on the small parameter $\nu$ is a common feature of strongly localized perturbations in 2D domains [45]. In Sect. 4 we determine corrections to the mean-field result (2.17) by solving the full steady-state Eq. (2.4) in the small cluster limit. These corrections take into account diffusion-mediated interactions between the clusters as well as the effects of the boundary $\partial \Omega$.

### 3D clustering model

**Fig. 3:**
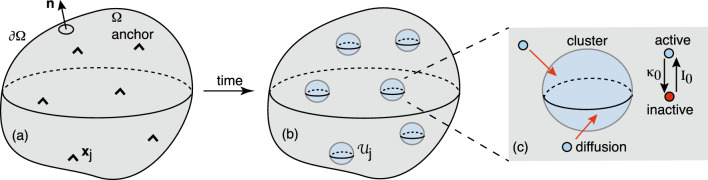
3D model of diffusion-based protein cluster formation in the presence of anchoring cites and particle activation/deactivation. **a** A set of *N* anchoring sites at positions ${\textbf{x}}_j$, $j=1,\ldots ,N$, in a bounded domain $\Omega$. **b** Diffusing particles in the activated state accumulate at the anchoring sites resulting in the formation of particles aggregates or clusters. **c** The clusters are dynamically maintained by a combination of diffusion and activation/deactivation

We now turn to the analogous problem of 3D particle clustering in the presence of anchoring sites that is maintained out-of-equilibrium by the activation/deactivation of diffusing particles; particles can only aggregate in the activated state, see Fig. 3. In particular, consider the mean field approximation given by Eq. (2.10) with $c(R)=0$ and the flux *J*(*R*) into the surface of each cluster determined self-consistently from the equation

2.18 $$\begin{aligned} J(R)=4\pi R^2 D\partial _rc(R). \end{aligned}$$

The solution of Eq. (2.10) in the 3D case is given by

2.19 $$\begin{aligned} c(r)=\left( c_0-\frac{\phi _0J(R)}{\kappa _0}\right) \left( 1-\frac{R}{r}\frac{{\mathrm e}^{-r/\lambda }}{{\mathrm e}^{-R/\lambda }}\right) . \end{aligned}$$

Substituting the solution into Eq. (2.18) yields a self consistency equation for the flux *J*(*R*):

2.20 $$\begin{aligned} J(R)=\frac{4\pi R^2D}{\lambda } \left( c_0-\frac{\phi _0J(R)}{\kappa _0}\right) \left[ 1+\frac{\lambda }{R}\right] . \end{aligned}$$

Equation (2.20) can be rewritten as an implicit equation for the cluster radius *R*, since Eq. (2.5) implies that $J(R)=4\pi R^3 \kappa _0 u_0/3$ [25]:

2.21 $$\begin{aligned} \frac{R}{\lambda }=3 \left( \frac{c_0}{u_0}-\frac{4\pi }{3}\left( \frac{R}{\lambda }\right) ^3 \phi _0\lambda ^3\right) \left[ 1+\frac{\lambda }{R}\right] . \end{aligned}$$

Finally, suppose that $R\ll \lambda$ and $4\phi _0 \pi \lambda ^3/3 \ll 1$. Setting $R/\lambda =\epsilon \rho /\lambda$, we find that

2.22 $$\begin{aligned} \left( \frac{\rho }{\lambda }\right) ^2&= \frac{3c_0}{\epsilon ^2 u_0}\left[ 1+\frac{\epsilon \rho }{\lambda }\right] . \end{aligned}$$

The rescaled radius is *O*(1) provided that we set $u_0={\overline{u}}_0/\epsilon ^3$ and $I_0={\overline{I}}_0/\epsilon$. Hence,

2.23 $$\begin{aligned} \rho&\approx \rho _0 \left[ 1+\frac{\epsilon \rho _0}{2\lambda }\right] , \end{aligned}$$

where

2.24 $$\begin{aligned} \rho _0\equiv \sqrt{\frac{3 \lambda ^2{\overline{c}}_0}{{\overline{u}}_0}}. \end{aligned}$$

In Sect. 5, we use matched asymptotics to determine corrections to the mean field result in the small cluster limit.

### Mean field theory of Ostwald ripening

It is useful to compare the 3D clustering model with the classical formulation of Oswald ripening during late-stage liquid-liquid phase separation. Suppose that we reinterpret Fig. 3 as a 3D domain $\Omega$ containing a set of *N* liquid droplets ${{\mathcal {U}}}_k$, $k=1,\ldots ,N$, that are well separated from each other and whose total volume fraction is relatively small. The concentration within each droplet is the high density phase $\phi _b$, whereas the concentration in the bulk domain is the low density phase $\phi _a$. The no-flux boundary condition on $\partial \Omega$ ensures mass conservation. Suppose that the coarsening dynamics is much slower than the equilibration of the concentration profile in the dilute phase. Under this quasi-static approximation, the solute concentration $\phi$ exterior to the droplets satisfies a steady-state diffusion equation of the form

2.25a $$\begin{aligned} \nabla ^2 \phi =0,\quad {\textbf{x}}\in \Omega \backslash \cup _{i=1}^N{{\mathcal {U}}}_{i},\quad \nabla \phi \cdot {\textbf{n}}=0 \text{ on } \partial \Omega ,\nonumber \\ \end{aligned}$$

and

2.25b $$\begin{aligned} \phi =\phi _a\left( 1+\frac{\ell _c}{R_i}\right) \equiv {\phi }_a(R_i) \text{ on } \partial {{\mathcal {U}}}_{i}. \end{aligned}$$

The boundary condition (2.25b) expresses the Gibbs–Thomson law due to interfacial tension on the droplet interface. The quasi-static approximation ensures that the total volume of condensates is conserved. This follows from integrating Eq. (2.25a) with respect to ${\textbf{x}}\in \Omega \backslash \cup _{i=1}^N{{\mathcal {U}}}_{i}$ and using the divergence theorem:

2.26 $$\begin{aligned} \sum _{j=1}^N \int _{{{\mathcal {U}}}_j}\nabla \phi ({\textbf{x}})\cdot {\textbf{n}}d{\textbf{x}}=0. \end{aligned}$$

That is, the sum of the fluxes into the *N* condensates is zero so that there is no net change in the total condensate volume.

**Fig. 4:**
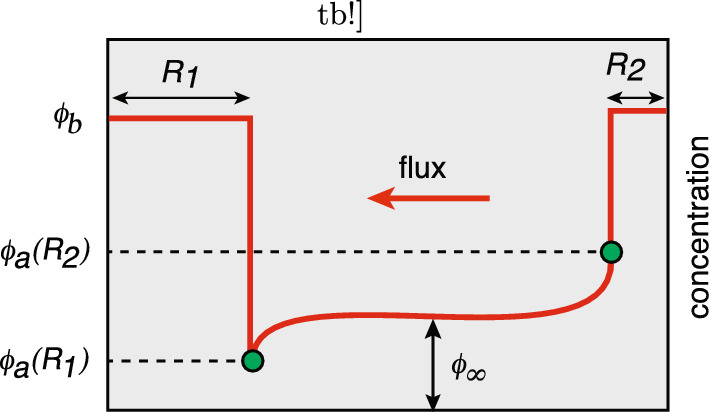
Ostwald ripening. Schematic diagram showing the concentration profile as a function of *x* along the axis joining the centers of two well separated droplets with different radii $R_1>R_2$. The solute concentration ${\phi }_a(R_1)$ around the larger droplet is lower than the concentration ${\phi }_a(R_2)$ around the smaller droplet, resulting in a net diffusive flux from the small droplet to the large droplet. Here $\phi _{\infty }$ denotes the mean-field of LSW theory

In the case of classical coarsening via Ostwald ripening, the difference in surface concentrations $\phi _a(R_i)$ for droplets of different sizes results in a net diffusive flux from small to large droplets. This is illustrated in Fig. 4 for two droplets ${{\mathcal {U}}}_1$ and ${{\mathcal {U}}}_2$ with $R_2 < R_1$. Under the mean field approximation, the effects of the boundary $\partial \Omega$ and interactions between droplets are ignored by introducing a constant mean field $\phi _{\infty }$ with $\phi ({\textbf{x}})\approx \phi _{\infty }$ for all ${\textbf{x}}\in \Omega$ such that $|{\textbf{x}}-{\textbf{x}}_i|\gg R_i$, $i=1,\ldots ,N$. The quantity $\Delta =\phi _{\infty }-\phi _a$ is known as the supersaturation, and is determined self-consistently from mass conservation. (In the absence of interfacial tension effects, the bulk volume fraction would simply be $\phi _a$ and, hence, $\Delta =0$.) Under the above assumptions, we can focus on the concentration outside a single droplet of radius *R*, which is analogous to the mean field approximation of clustering. Using spherical polar coordinates, we have

2.27 $$\begin{aligned} 0=\frac{D}{r^2}\frac{\partial }{\partial r}\left[ r^2\frac{\partial \phi }{\partial r}\right] ,\quad r>R, \end{aligned}$$

together with the boundary conditions

2.28 $$\begin{aligned} \phi (R)= {\phi }_a(R),\quad \phi (r)\rightarrow \phi _{\infty } \text{ as } r \rightarrow \infty . \end{aligned}$$

Hence,

2.29 $$\begin{aligned} \phi (r)=\phi _{\infty }-\frac{R}{r}\left( \Delta -\frac{\phi _a\ell _c}{R}\right) , \end{aligned}$$

which implies that the diffusive flux entering the droplet at its interface is

2.30 $$\begin{aligned} J(R)=D\phi '(R)=\frac{D}{R}\left( \Delta -\frac{\phi _a\ell _c}{R}\right) . \end{aligned}$$

It follows that $R_c= \phi _a \ell _c/\Delta$ is a critical radius such that $J(R)>0$ ($J(R) <0$) when $R>R_c$ ($R<R_c$),which means that the droplet grows (shrinks) on longer time scales.

Using a separation of time scales, one can now write down dynamical equations for the evolution of the droplet radii. This is driven by the transfer of solute molecules between the dilute and dense phases as determined by the flux. When the radius $R_j$ increases by an amount $dR_j$, the area increases by $dA_j=4\pi R_j^2dR_j$ and the number of molecules required to enlarge the droplet by an amount $dR_j$ is $\phi _bdA_j$ (assuming for simplicity that $\phi _b \gg \phi (R_j)$). These molecules are supplied by the flux at the interface. One thus finds that

2.31 $$\begin{aligned} \frac{dR_j}{\text {d}t} =\frac{J(R_j)}{\phi _b}=\frac{\Gamma }{R_j}\left( \frac{1}{R_c}-\frac{1}{R_j}\right) ,\quad j=1,\ldots ,N,\nonumber \\ \end{aligned}$$

where

2.32 $$\begin{aligned} \Gamma = \frac{D\phi _a\ell _c}{ \phi _b}. \end{aligned}$$

Multiplying both sides of Eq. (2.31) by $R_j^2$, summing over *j*, and imposing conservation of the total droplet volume $V_\textrm{drop} =4\pi \sum _jR_j^3(t)/3$ gives

2.33 $$\begin{aligned} R_c(t)=\frac{1}{N}\sum _{j=1}^NR_j(t). \end{aligned}$$

It follows that

2.34 $$\begin{aligned} \Delta (t)\equiv \phi _{\infty }(t)-\phi _a=\frac{\ell _c\phi _a N}{\sum _{j=1}^NR_j(t)}. \end{aligned}$$

Equation (2.34) implies that $\phi _{\infty }(t)$ decreases as the mean radius increases. Since the critical radius $R_c(t)$ increases as the saturation $\Delta (t)=\phi _{\infty }(t)-\phi _a$ decreases, it follows that only a single droplet remains in the limit $t\rightarrow \infty$.

As we have discussed in detail elsewhere [42–44], mean field theory becomes less accurate in the case of circular droplets in 2D systems, since the concentration around a droplet varies as $\ln R$ rather than $R^{-1}$, where *R* is the distance from the center of the droplet. Thus, more care must be taken in imposing far-field conditions, as previously shown for classical Ostwald ripening [56, 57]. Mean field theory also fails to capture finite-size effects. Corrections to mean field theory based on asymptotic methods are briefly considered in Sect. 3.3 for both 2D and 3D droplets.

## Diffusion in a singularly perturbed domains

In this section, we review the basic theory of diffusion in singularly perturbed domains by considering the bounded domain $\Omega \subset {{\mathbb {R}}}^d$ with *N* small interior subdomains ${{\mathcal {U}}}_j$, as shown in Fig. 1b for 2D and Fig. 3b for 3D. However, in contrast to the models of clustering and droplet formation described in Sect. 2, we consider a simpler steady-state BVP in which the radius $R_j$ of the subdomain ${{\mathcal {U}}}_j$ is fixed and is known *a priori*. Let $c({\textbf{x}})$ denote the particle concentration exterior to the subdomains ${{\mathcal {U}}}_j$, and suppose that the concentration on the boundary $\partial {{\mathcal {U}}}_j$ has a fixed value $c_j$:

3.1a $$\begin{aligned} {\varvec{\nabla }}^2 c=0,\quad {\textbf{x}}\in \Omega \backslash \cup _{i=1}^N{{\mathcal {U}}}_{i}, \end{aligned}$$

supplemented by the boundary conditions

3.1b $$\begin{aligned} {\textbf{n}}\cdot {\varvec{\nabla }}c=0 \text{ on } \partial \Omega , \quad c=c_j \text{ on } \partial {{\mathcal {U}}}_{j}. \end{aligned}$$

We leave the physical interpretation of ${{\mathcal {U}}}_j$ open here, and focus on the mathematical analysis. In classical Ostwald ripening, ${{\mathcal {U}}}_j$ is a liquid droplet, $c_j= \phi _a(R_j)$, and $R_j$ is time-dependent. On the other hand, in the clustering model, ${{\mathcal {U}}}_j$ is an anchored cluster whose radius is determined self-consistently from the interfacial fluxes. We also note that Eq. (2.4) can be rewritten in a form similar to Eq. (3.1) by performing the shift $c({\textbf{x}},t)\rightarrow c'({\textbf{x}},t)=c({\textbf{x}},t)-c_0$ with $c_0= I_0/\kappa _0$. After dropping the ^′^ on $c'$, we have

3.2a $$\begin{aligned}&D{\varvec{\nabla }}^2 c({\textbf{x}},t)-\kappa _0 c({\textbf{x}},t)=0,\ {\textbf{x}}\in \Omega \backslash \cup _{j=1}^N {{\mathcal {U}}}_j(t), \end{aligned}$$

3.2b $$\begin{aligned}&D{\varvec{\nabla }} c({\textbf{x}},t) \cdot {\textbf{n}}=0,\ {\textbf{x}}\in \partial \Omega ,\end{aligned}$$

3.2c $$\begin{aligned}&c({\textbf{x}},t)=-c_0,\quad {\textbf{x}}\in \partial {{\mathcal {U}}}_j(t). \end{aligned}$$

**Fig. 5:**
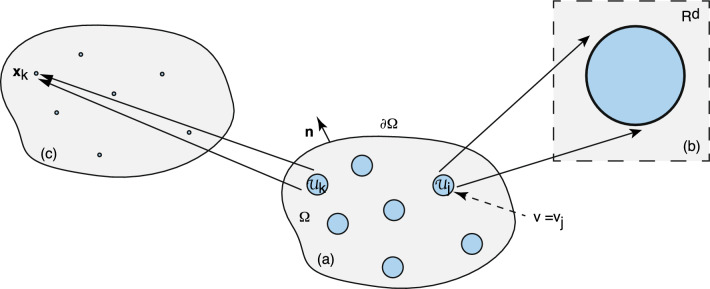
Diffusion in a singularly perturbed domain. **a** Particles diffuse in a bounded domain $\Omega$ containing *N* small interior holes or perforations denoted by ${{\mathcal {U}}}_j$, $j=1,\ldots ,N$. The exterior boundary $\partial \Omega$ is reflecting, whereas $c({\textbf{x}})=c_j$ on the *j*-th interior boundary $\partial {{\mathcal {U}}}_j$. **b** Construction of the inner solution in terms of stretched coordinates ${\textbf{y}}=\epsilon ^{-1}({\textbf{x}}-{{\textbf{x}}}_j)$, where ${{\textbf{x}}}_j$ is the center of the *j*-th hole. The rescaled radius is $\rho _j$ and the region outside the hole is taken to be ${{\mathbb {R}}}^d$, $d=2,3$, rather than the bounded domain $\Omega$. **c** Construction of the outer solution. Each hole is shrunk to a single point. The outer solution can be expressed in terms of the corresponding modified Neumann Green’s function and then matched with the inner solution around each hole

The basic assumption of the asymptotic method is that the subdomains ${{\mathcal {U}}}_j$ are small and well separated. Fixing the length scale by setting $L:=|\Omega |^{1/2}=1$, we then take $R_j=\epsilon \rho _j$ with $0<\epsilon \ll 1$, $|{\textbf{x}}_i-{\textbf{x}}_j| =O(1)$ for all $j\ne i$ and $\min _{{\textbf{y}}}\{|{\textbf{x}}_j -{\textbf{y}}|,{\textbf{y}}\in \partial \Omega \} =O(1)$, $j=1,\ldots ,N$. Under these various conditions, we can use a combination of matched asymptotics and Green’s function methods along analogous lines to Refs. [45–54]. More specifically, we construct an inner or local solution valid in an $O(\epsilon )$ neighborhood of each cluster, and then match to an outer or global solution that is valid away from each neighborhood. The general construction is illustrated in Fig. 5. We now give a detailed description of the steps of the analysis, first in 2D and then 3D.

### Asymptotic analysis in 2D

*(i) Inner solution.* The inner solution in a neighborhood of ${{\mathcal {U}}}_j$ is defined by introducing the stretched coordinates ${\textbf{y}}=\epsilon ^{-1}({\textbf{x}}-{\textbf{x}}_j)$, replacing the domain $\Omega$ by ${{\mathbb {R}}}^2$, see Fig. 5b, and setting $C_j({\textbf{y}})=c({\textbf{x}}_j+\epsilon {\textbf{y}})$. One can view this procedure as zooming into the subdomain ${{\mathcal {U}}}_j$ and ignoring the effects of the boundary $\partial \Omega$ and the other subdomains ${{\mathcal {U}}}_k$, $k\ne j$. It follows that

3.3a $$\begin{aligned} \nabla _{{\textbf{y}}}^2 C_j({\textbf{y}})&=0 \text{ for } {\textbf{y}}\in {{\mathbb {R}}}^2\backslash {{\mathcal {U}}}_j, \end{aligned}$$

3.3b $$\begin{aligned} C_j({\textbf{y}})&= c_j \text{ on } |{\textbf{y}}|=\rho _j, \end{aligned}$$

which can be expressed in polar coordinates as

3.4 $$\begin{aligned}&\frac{1}{\rho }\frac{d}{d\rho }\rho \frac{dC_j(\rho )}{d\rho }=0,\quad \rho _j< \rho <\infty , \quad C_j(\rho _i)=c_j . \end{aligned}$$

The solution takes the form

3.5 $$\begin{aligned} C_j(\rho )=c_j+ A_j \ln ( \rho /\rho _j),\quad \rho >\rho _j, \end{aligned}$$

where $A_i$ is some undetermined coefficient. The corresponding solution in the original coordinates is

3.6 $$\begin{aligned} C_j({\textbf{x}})=c_j+ A_j \ln ( |{\textbf{x}}-{\textbf{x}}_j| /\epsilon \rho _j). \end{aligned}$$

The coefficients $$A_j $$, $$j=1,\ldots ,N$$, can be determined by matching the inner solutions with the corresponding outer solution (see below).

*(ii) Outer solution.* The outer solution is obtained by treating ${{\mathcal {U}}}_j$ as a point source/sink, see Fig. 5c. This is equivalent to zooming out of the domain $\Omega$. The resulting diffusion equation takes the form

3.7a $$\begin{aligned} {\varvec{\nabla }}^2 c&=0,\quad {\textbf{x}}\in \Omega \backslash \{{\textbf{x}}_1,\ldots ,{\textbf{x}}_N\}, \end{aligned}$$

3.7b $$\begin{aligned} { \textbf{n}}\cdot {\varvec{\nabla }} c&=0,\quad {\textbf{x}}\in \partial \Omega , \end{aligned}$$

together with the matching condition

3.7c $$\begin{aligned} c \sim c_j+\frac{A_j}{\nu }+ A_j \ln ( |{\textbf{x}}-{\textbf{x}}_j|/\rho _j), \ \nu =-\frac{1}{\ln \epsilon },\nonumber \\ \end{aligned}$$

as ${\textbf{x}}\rightarrow {\textbf{x}}_j$. The next step is to introduce the 2D Neumann Green’s function $G_0({\textbf{x}},{\textbf{y}})$, which is uniquely defined by

3.8a $$\begin{aligned} D{\varvec{\nabla }}^2 G_0&=\frac{1}{|\Omega |}-\delta ({\textbf{x}}-{\textbf{y}}),\quad {\textbf{x}}\in \Omega ; \end{aligned}$$

3.8b $$\begin{aligned} {\textbf{n}}\cdot {\varvec{\nabla }} G_0&=0 \text{ on } \partial \Omega ,\quad \int _{\Omega }G_0 d{\textbf{x}}=0 \end{aligned}$$

for fixed $${\textbf{y}}$$. (More precisely, $$G_0$$ is a modified Green’s function, since the solution of Laplace’s equation in a bounded domain $$\Omega $$ with a reflecting boundary $$\partial \Omega $$ is only defined up to a constant. This is the reason why there is the additional constant term $$1/\Omega $$ on the right-hand side of Eq. (3.8a). Indeed, integrating the left-hand side of Eq. (3.8a) with respect to $${\textbf{x}}\in \Omega $$ and imposing the Neumann boundary condition yields zero. This is consistent with the right-hand side as $$\int _{\Omega }[|\Omega |^{-1} -\delta (x-y)]d{\textbf{x}}=0$$. The additional condition $$\int _{\Omega }G_0 d{\textbf{x}}=0$$ determines $$G_0$$ uniquely. Note that there is no arbitrary constant in the construction of the Green’s function $$G({\textbf{x}},{\textbf{y}})$$ of the modified Helmholtz equation, see Eq. (2.7), where $${\varvec{\nabla }}^2G_0$$ is replaced by $${\varvec{\nabla }}^2G-\kappa _0G$$.) Finally, note that $$G_0 $$ can be decomposed as

3.9 $$\begin{aligned} G_0({\textbf{x}},{\textbf{y}})=-\frac{ \ln |{\textbf{x}}-{\textbf{y}}|}{2\pi }+{{\mathcal {R}}}_0({\textbf{x}},{\textbf{y}}), \end{aligned}$$

where ${{\mathcal {R}}}_0$ is the regular (non-singular) part of the Green’s function.

We now make the ansatz

3.10 $$\begin{aligned} c({\textbf{x}})\sim c_{\infty }-2\pi D \sum _{j=1}^NA_j G_0({\textbf{x}},{\textbf{x}}_j) \end{aligned}$$

for ${\textbf{x}}\notin \{{\textbf{x}}_j,\, j=1,\ldots ,N\}$ and some constant $c_{\infty }$. Using the fact that $\int _{\Omega } G_0d{\textbf{x}}=0$, it follows from Eq. (3.10) that

$$\begin{aligned} c_{\infty }=|\Omega |^{-1}\int _{\Omega }c({\textbf{x}})d{\textbf{x}}. \end{aligned}$$

We also observe that for ${\textbf{x}}\notin \{{\textbf{x}}_j,\, j=1,\ldots ,N\}$,

$$\begin{aligned} {\varvec{\nabla }}^2 c({\textbf{x}})&\sim -2\pi D \sum _{j=1}^NA_j {\varvec{\nabla }}^2 G_0({\textbf{x}},{\textbf{x}}_j)\\&= -\frac{2\pi }{|\Omega |} \sum _{j=1}^NA_j. \end{aligned}$$

Hence, the outer solution satisfies the steady-state diffusion equation if and only if

3.11 $$\begin{aligned} \sum _{j=1}^NA_j=0. \end{aligned}$$

*(iii) Matched asymptotics.* In order to determine the $N+1$ unknown coefficients, $A_j$, $j=1,\ldots ,N$ and $c_{\infty }$, we require $N+1$ linearly independent conditions. One of these is given by Eq. (3.11) whereas the others are obtained from the matching conditions (3.7c). The outer solution (3.10) shows that as ${\textbf{x}}\rightarrow {\textbf{x}}_j$,

3.12 $$\begin{aligned} c({\textbf{x}})&\rightarrow c_{\infty } +A_j \ln |{\textbf{x}}-{\textbf{x}}_j|-2\pi D A_j {{\mathcal {R}}}_0({\textbf{x}}_j,{\textbf{x}}_j)\nonumber \\&\quad -2\pi D\sum _{i\ne j} A_iG_0({\textbf{x}}_j,{\textbf{x}}_i). \end{aligned}$$

Comparison with the asymptotic limit in Eq. (3.7c) yields the self-consistency conditions

3.13 $$\begin{aligned} c_{\infty }-c_j&=\left[ \frac{1}{\nu } -\ln \rho _j + 1 +2\pi D {{\mathcal {R}}}_0({\textbf{x}}_j,{\textbf{x}}_j)\right] A_j\nonumber \\&\quad +2\pi D \sum _{i \ne j }^N A_i G_0({\textbf{x}}_j,{\textbf{x}}_i) \end{aligned}$$

for $j=1,\ldots ,N$. This is a matrix equation that can be inverted to give

3.14 $$\begin{aligned} A_i=A_i(\nu )\equiv \nu \sum _{j=1}^N[1+2\pi \nu D {{\mathcal {G}}}_0]^{-1}_{ij}\left( c_{\infty }-c_j\right) ,\nonumber \\ \end{aligned}$$

where

3.15 $$\begin{aligned} {{\mathcal {G}}}_{0,jj}&={{\mathcal {R}}}_0 ({\textbf{x}}_j,{\textbf{x}}_j)-\frac{\ln \rho _j}{2\pi D} \text{ and } {{\mathcal {G}}}_{0,ji}=G_0({\textbf{x}}_j,{\textbf{x}}_i) \end{aligned}$$

for $j\ne i$. It remains to determine the unknown constant $c_{\infty }$. Imposing the constraint (3.11) on Eq. (3.14) implies that

3.16 $$\begin{aligned} c_{\infty }&=\left[ {\displaystyle \sum _{i,j=1}^N {{\mathcal {M}}}_{ij}}\right] ^{-1} {\displaystyle \sum _{i,j=1}^N {{\mathcal {M}}}_{ij}c_j}. \end{aligned}$$

where

3.17 $$\begin{aligned} {{\mathcal {M}}}=[1+2\pi \nu D {{\mathcal {G}}}_0]^{-1}. \end{aligned}$$

Finally, note that the solution (3.14) is a non-perturbative function of the small parameter $\nu$. This was achieved by matching the inner and outer solutions using Green’s functions along the lines originally developed in Refs. [45, 46]. This effectively sums over the logarithmic terms, which is equivalent to calculating the asymptotic solution for all terms of $O(\nu ^k)$ for any *k*. Obtaining a non-perturbative solution is important, since $\nu \rightarrow 0$ much more slowly than $\epsilon \rightarrow 0$, and is a characteristic feature of diffusion problems in 2D singularly perturbed domains.

### Asymptotic analysis in 3D

The analysis in 3D is based on an asymptotic expansion with respect to $\epsilon$ rather than $\nu$. The outer solution is expanded as

3.18 $$\begin{aligned} c({\textbf{x}})\sim c_{\infty }+\epsilon c_1({\textbf{x}})+\epsilon ^2 c_2({\textbf{x}})+\ldots \end{aligned}$$

with

3.19a $$\begin{aligned} D{\varvec{\nabla }}^2 c_{\ell }({\textbf{x}}) =&0,\quad {\textbf{x}}\in \Omega \backslash \{{\textbf{x}}_1,\ldots ,{\textbf{x}}_N\}, \end{aligned}$$

3.19b $$\begin{aligned} {\varvec{\nabla }}c_{\ell }({\textbf{x}})\cdot {\textbf{n}}&=0,\quad {\textbf{x}}\in \partial \Omega , \end{aligned}$$

for $\ell =1,2.\ldots$, together with certain singularity conditions as ${\textbf{x}}\rightarrow {\textbf{x}}_j$, $j=1,\ldots ,N$. The latter are determined by matching to the inner solution. In the inner region around the *j*-th cluster, we again introduce the stretched coordinates $\textbf{y}=\epsilon ^{-1}({\textbf{x}}-{\textbf{x}}_j)$ and set $C_j(\textbf{y}) =c({\textbf{x}}_j+\epsilon {\textbf{y}})$. Expanding the inner solution as

3.20 $$\begin{aligned} C_j({\textbf{y}}) = C_{j,0}({\textbf{y}}) +\epsilon C_{j,1}({\textbf{y}})+\ldots , \end{aligned}$$

we have

3.21 $$\begin{aligned} {\varvec{\nabla }}_\textbf{y}^2 C_{j,\ell }&=0, \quad {\textbf{y}}\in {{\mathbb {R}}}^3\backslash {{\mathcal {U}}}_j \end{aligned}$$

3.22 $$\begin{aligned} C_{j,\ell }({\textbf{y}})&=c_j\delta _{\ell ,0}, \quad {\textbf{y}}\in \partial {{\mathcal {U}}}_j. \end{aligned}$$

Finally, the matching condition is that the near-field behavior of the outer solution as ${\textbf{x}}\rightarrow {\textbf{x}}_j$ should agree with the far-field behavior of the inner solution as $|{\textbf{y}}|\rightarrow \infty$, which is expressed as

3.23 $$\begin{aligned} c_{\infty }+ \epsilon c_1({\textbf{x}})+\epsilon ^2 c_2({\textbf{x}}) \sim C_{j,0}({\textbf{x}})+ \epsilon C_{j,1}({\textbf{x}})+\ldots \nonumber \\ \end{aligned}$$

In particular, note that the far-field behavior of $C_{j,\ell }$ determines the near-field behavior of $c_{j,\ell +1}$ so we alternate between the inner and outer solutions during matching.

First $C_{j,0} \sim c_{\infty }$ as $|{\textbf{y}}|\rightarrow \infty$ so that we can set

3.24 $$\begin{aligned} C_{j,0}({\textbf{y}}) =c_jw({\textbf{y}})+c_{\infty }(1-w({\textbf{y}})), \end{aligned}$$

with $w({\textbf{y}})$ satisfying the boundary value problem

3.25 $$\begin{aligned} {\varvec{\nabla }}_\textbf{y}^2 w({\textbf{y}})&=0,\ {\textbf{y}}\in {{\mathbb {R}}}^3\backslash {{\mathcal {U}}}_j ; \quad w({\textbf{y}})=1,\ {\textbf{y}}\in \partial {{\mathcal {U}}}_j,\nonumber \\ w({\textbf{y}})&\rightarrow 0\quad \text{ as } |{\textbf{y}}|\rightarrow \infty . \end{aligned}$$

This is a well-known problem in electrostatics and for a spherical subdomain has the exact solution

3.26 $$\begin{aligned} w({\textbf{y}})=\frac{\rho _j}{\rho }. \end{aligned}$$

(See the discussion for the generalization to non-spherical shapes.) The matching condition (3.23) then implies that $c_1({\textbf{x}})$ satisfies Eq. (3.19) together with the singularity condition

$$\begin{aligned}c_1({\textbf{x}})\sim -\frac{[c_{\infty }-c_j] \rho _j}{|{\textbf{x}}-{\textbf{x}}_j|} \quad \text{ as } {\textbf{x}}\rightarrow {\textbf{x}}_j.\end{aligned}$$

It follows that the solution can be written as

3.27 $$\begin{aligned} c_1({\textbf{x}})=-{4\pi } D \sum _{k=1}^N\rho _k[c_{\infty }-c_k]G_0({\textbf{x}},{\textbf{x}}_k), \end{aligned}$$

where $G_0$ is the 3D version of the Green’s function defined in Eq. (3.8). In particular, the 3D Green’s function has the singularity structure

3.28 $$\begin{aligned} G_0({\textbf{x}},{\textbf{x}}')=\frac{1}{4\pi D|{\textbf{x}}-{\textbf{x}}'|} +{{\mathcal {R}}}_0({\textbf{x}},{\textbf{x}}'). \end{aligned}$$

Note that for ${\textbf{x}}\notin \{{\textbf{x}}_j,\, j=1,\ldots ,N\}$,

$$\begin{aligned} \nabla ^2 c_1({\textbf{x}})&\approx -4\pi \sum _{i=1}^N \rho _i[c_{\infty }-c_i]{\varvec{\nabla }}^2 G_0({\textbf{x}},{\textbf{x}}_i)\\&=-\frac{4\pi }{|\Omega |} \sum _{i=1}^N\rho _i[c_{\infty }-c_i] \end{aligned}$$

Hence, the $O(\epsilon )$ term in the expansion of the outer solution satisfies the steady-state diffusion equation if and only if $\sum _{i=1}^N\rho _i[c_{\infty }-c_i]=0$, which can be rearranged to show that the mean field

3.29 $$\begin{aligned} c_{\infty }=\frac{ \sum _{i=1}^N\rho _i c_i}{ \sum _{i=1}^N\rho _i }. \end{aligned}$$

Next we match the far-field behavior of $C_{j,1}({\textbf{y}})$ with the near-field behavior of $c_1({\textbf{x}})$ around ${{\mathcal {U}}}_j$, which takes the form

3.30 $$\begin{aligned} c_1({\textbf{x}})&\sim \frac{[ c_j-c_{\infty }]\rho _j}{|{\textbf{x}}-{\textbf{x}}_j|}+[ c_j-c_{\infty }]\chi _j, \end{aligned}$$

with

3.31 $$\begin{aligned} \chi _j&=4\pi D \rho _j {{\mathcal {R}}}({\textbf{x}}_j,{\textbf{x}}_j)+ 4\pi D \sum _{k\ne j}^N\rho _k G_0({\textbf{x}}_j,{\textbf{x}}_k). \end{aligned}$$

It follows that the solution of Eq.(3.21) for $\ell =1$ is

3.32 $$\begin{aligned} C_{j,1}({\textbf{y}})=-[c_{\infty }-c_j] \chi _ j( 1-w({\textbf{y}})),\ \end{aligned}$$

with $w({\textbf{y}})$ given by Eq. (3.25). Hence, $c_2$ satisfies Eq. (3.19) supplemented by the singularity condition

$$\begin{aligned} c_2({\textbf{x}})\sim \frac{[c_{\infty }-c_j] \chi _j\rho _j}{|{\textbf{x}}-{\textbf{x}}_j|} \quad \text{ as } {\textbf{x}}\rightarrow {\textbf{x}}_j. \end{aligned}$$

Following along identical lines to the derivation of $c_1({\textbf{x}})$, we obtain the result

3.33 $$\begin{aligned} c_2({\textbf{x}})=4\pi D\sum _{k=1}^N[c_{\infty }-c_k] \chi _k\rho _kG_0({\textbf{x}},{\textbf{x}}_k). \end{aligned}$$

In conclusion, the outer solution takes the form

3.34 $$\begin{aligned} c({\textbf{x}}) \sim c_{\infty } \!-\!{4\pi } D \sum _{k=1}^N[c_{\infty }\!-\! c_k] \rho _k(1\!-\!\epsilon \chi _k)G_0({\textbf{x}},{\textbf{x}}_k) ,\nonumber \\ \end{aligned}$$

while the inner solution around the *j*-th cluster is

3.35 $$\begin{aligned} C_j(\rho )\sim c_{\infty }\!-\! (c_{\infty }\!-\!c_j)\left( \frac{ \rho _j}{\rho }+ \epsilon \chi _j \left( 1\!-\!\frac{ \rho _j}{\rho }\right) \right) .\nonumber \\ \end{aligned}$$

### Droplet kinetics during Ostwald ripening

The asymptotic expansions derived in Sects. 3.1 and 3.2 can be applied directly to the classical model of Ostwald ripening described in Sect. 2.4. First, we set $c({\textbf{x}})=\phi ({\textbf{x}})$, $c_{\infty }=\phi _{\infty }$, and take

3.36 $$\begin{aligned} c_j={\phi }_a(R_i) \equiv \phi _a\left( 1+\frac{\ell _c}{R_i}\right) . \end{aligned}$$

It follows that

3.37 $$\begin{aligned} c_{\infty }-c_j=\Delta - \frac{\ell _c\phi _a}{R_i}, \end{aligned}$$

where $\Delta =\phi _{\infty }-\phi _a$ is known as the supersaturation. Given the choice of scaling with $R_j=\epsilon \rho _j$ we also assume that $\ell _c=\epsilon \ell _c'$.

Let us begin with the simpler case of 3D droplets considered in Sect. 3.3. Equation (2.31) still holds under the identification

3.38 $$\begin{aligned} J(R_i)=\frac{D}{\epsilon } \Phi '(\rho _i) \end{aligned}$$

where $\Phi (\rho _i)$ is the inner solution obtained from Eq. (3.35) for a spherical droplet:

3.39 $$\begin{aligned} \Phi _j(\rho ) \sim c_{\infty }-\left( \Delta - \frac{\ell _c'\phi _a}{\rho _j}\right) \left( \frac{ \rho _j}{\rho }+ \epsilon \chi _j \left( 1-\frac{ \rho _j}{\rho }\right) \right) \nonumber \\ \end{aligned}$$

for $\rho >\rho _j$ and $\chi _j$ given by Eq. (3.31). The supersaturation $\Delta$ is determined from Eq. (3.29), which yields

3.40 $$\begin{aligned} \Delta =\frac{\ell _c'\phi _a N}{\sum _{j=1}^N\rho _j}= \frac{\ell _c\phi _a N}{\sum _{j=1}^NR_j}. \end{aligned}$$

We thus recover the classical result (2.34). This is not surprising, in the sense that Eq. (3.29) reflects conservation of the total droplet volume. Finally, substituting for $J(R_i)$ into the kinetic Eq. (2.31) with $R_j=\epsilon \rho _j$ gives

3.41 $$\begin{aligned} \epsilon ^2 \frac{d\rho _j}{\text {d}t}&=\frac{D\Phi '(\rho _j)}{\phi _b}\nonumber \\&=\frac{D}{\phi _b \rho _j}\left( \Delta - \frac{\ell _c'\phi _a}{\rho _j}\right) \left( 1-\epsilon \chi _j \right) \nonumber \\&=\frac{\Gamma }{\rho _j}\left( \frac{1}{\rho _c}-\frac{1}{\rho _j}\right) \left( 1-\epsilon \chi _j \right) \end{aligned}$$

for $$j=1,\ldots ,N$$, where

3.42 $$\begin{aligned} \Gamma = \frac{D\phi _a\ell '_c}{ \phi _b},\quad \rho _c=\frac{1}{N}\sum _{j=1}\rho _j. \end{aligned}$$

This establishes how the asymptotic analysis generates corrections to the mean field droplet kinetics.

In the case of 2D droplets, there is no mean field limit. However, we can still use the asymptotic analysis to derive kinetic equations for the droplet sizes. The inner solution (3.6) near the *j*-th droplet takes the form

3.43 $$\begin{aligned} \Phi _j(\rho )\sim \phi _a\left( 1+\frac{\ell _c'}{\rho _j} \right) +\nu \left( \Delta -\frac{\phi _a\ell _c'}{\rho _j}\right) \ln (\rho /\rho _i).\nonumber \\ \end{aligned}$$

For the sake of illustration, we have expanded the coefficient $A_i$ in Eq. (3.14) to leading order in $\nu$. The analogous expansion of the condition (3.16) with ${{\mathcal {M}}}_{ij}\sim \delta _{i,j}+O(\nu )$ shows that to leading order in $\nu$,

3.44 $$\begin{aligned} \Delta \sim \frac{1}{N}\sum _{i=1}^N\frac{\phi _a}{\rho _i}=\frac{\phi _a}{\rho _\textrm{harm}}, \end{aligned}$$

where $\rho _\textrm{harm}$ is the harmonic mean [42, 57]

3.45 $$\begin{aligned} \frac{1}{\rho _\textrm{harm}}=\frac{1}{N}\sum _{j=1}^N\frac{1}{\rho _j}. \end{aligned}$$

This establishes one major difference from 3D, where $\Delta$ is given by the inverse of the arithmetic mean of the radii, see Eq. (3.40).

Given the quasi-static solution for the concentration, we can now write down a dynamical equation for the rate of change of the size of each circular droplet along analogous lines to the 3D case. When the radius $R_i$ increases by an amount $dR_i$, the area increases by $dA_i=2\pi R_i dR_i$ and the number of molecules required to enlarge the droplet by an amount $dR_i$ is $\phi _bdA_i$ (assuming for simplicity that $\phi _b \gg \phi (R_i)$). These molecules are supplied by the flux at the interface. Hence, we have

3.46 $$\begin{aligned} \epsilon ^2\frac{d\rho _i}{\text {d}t}&= \frac{D}{\phi _b } \Phi _i'(\rho _i)\approx \frac{D}{\phi _b} \frac{\nu }{ \rho _i}\left( \Delta -\frac{\phi _a}{\rho _i}\right) . \end{aligned}$$

In contrast to 3D, there is no *O*(1) contribution on the right-hand side, reflecting the breakdown of mean field theory.

## Asymptotic analysis of the 2D clustering model

In order to apply the theory developed in Sect. 3.1, we use the version of the clustering model given by Eq. (3.2). It follows that Eq. (3.1a) becomes ${\varvec{\nabla }}^2 c-\kappa _0c=0$ while the boundary condition (3.1b) still holds with $c_j=-c_0$ for all *j*. First, consider the inner equation. Introducing stretched coordinates around ${{\mathcal {U}}}_j$, we have in polar coordinates

4.1a $$\begin{aligned}&\frac{1}{\rho }\frac{d}{d\rho }\rho \frac{dC_j(\rho )}{d\rho }-\epsilon ^2 \kappa _0 C_j(\rho )=0,\quad \rho >\rho _j , \end{aligned}$$

4.1b $$\begin{aligned} C_j(\rho _i)&=-c_0 . \end{aligned}$$

Since we are only working to leading order in $\epsilon$ (but to all orders in $\nu$), we can drop the $\epsilon ^2 \kappa _0 C_j$ term, which recovers the inner problem given by Eq. (3.4). Hence, the inner solution is

4.2 $$\begin{aligned} C_j(\rho )=-c_0+ A_j \ln ( \rho /\rho _j),\quad \rho >\rho _j. \end{aligned}$$

The corresponding outer equation takes the form

4.3a $$\begin{aligned} D{\varvec{\nabla }}^2 c({\textbf{x}})-\kappa _0c({\textbf{x}}) =&0,\quad {\textbf{x}}\in \Omega \backslash \{{\textbf{x}}_1,\ldots ,{\textbf{x}}_N\}, \end{aligned}$$

4.3b $$\begin{aligned} {\varvec{\nabla }}c({\textbf{x}})\cdot {\textbf{n}}&=0,\quad {\textbf{x}}\in \partial \Omega , \end{aligned}$$

together with the matching condition

4.3c $$\begin{aligned} c({\textbf{x}})\sim \frac{A_j}{\nu }+ A_j \ln (|{\textbf{x}}-{\textbf{x}}_j|/\rho _j) \end{aligned}$$

as ${\textbf{x}}\rightarrow {\textbf{x}}_j$. The outer solution (3.10) becomes

4.4 $$\begin{aligned} c({\textbf{x}})\sim -2\pi D \sum _{j=1}^NA_j G({\textbf{x}},{\textbf{x}}_j) \end{aligned}$$

for ${\textbf{x}}\notin \{{\textbf{x}}_j,\, j=1,\ldots ,N\}$ where $G({\textbf{x}},{\textbf{y}})$ is the Green’s function of the modified Helmholtz equation, see Eq. (2.7). Using the identity $\int _{\Omega }G({\textbf{x}},{\textbf{x}}')d{\textbf{x}}=1/\kappa _0$ we see from Eq. (4.4) that

4.5 $$\begin{aligned} \int _{\Omega }c({\textbf{x}})d{\textbf{x}}\sim -\frac{2\pi D}{\kappa _0}\sum _{j=1}A_j. \end{aligned}$$

We can now determine the unknown coefficients $$A_j $$, $$j=1,\ldots ,N$$, by matching the inner and outer solutions along identical lines to Sect. 3.1. We thus obtain the solution

4.6 $$\begin{aligned} A_i =A_i(\nu )\equiv \nu c_0\sum _{j=1}^N[1+2\pi \nu D {{{\mathcal {G}}}}]^{-1}_{ij}, \end{aligned}$$

with

4.7 $$\begin{aligned} {{{\mathcal {G}}}}_{jj}&={{\mathcal {R}}}({\textbf{x}}_j,{\textbf{x}}_j)-\frac{\ln \rho _j}{2\pi D} ,\quad {{{\mathcal {G}}}}_{ji}=G({\textbf{x}}_j,{\textbf{x}}_i) \end{aligned}$$

for $j\ne i$. In order to calculate the coefficients $A_i(\nu )$ we need to obtain accurate numerical or analytical approximations of the Green’s function for the modified Helmholtz equation and inverting the matrix in E. (4.6). This particular issue has been addressed by Lindsay *et al.* [53], whose results can be applied to the current problem. An important step in the evaluation of the Green’s function is to decompose *G* as the sum of the free-space Green’s function and a regular part:

4.8 $$\begin{aligned} G({\textbf{x}},{\textbf{x}}') = \frac{1}{2\pi D}K_0\left( |{\textbf{x}}- {\textbf{x}}'|/\lambda \right) + {\widehat{{{\mathcal {R}}}}}({\textbf{x}},{\textbf{x}}'), \end{aligned}$$

where $\lambda =\sqrt{D/\kappa _0}$, $K_0$ is the modified Bessel function of the second kind and ${\widehat{{{\mathcal {R}}}}}$ is non-singular at ${\textbf{x}}={\textbf{x}}'$. It can be shown that for $|{\textbf{x}}- {\textbf{x}}'| = O(1)$ and sufficiently small $\lambda$, the boundary contributions to ${\widehat{{{\mathcal {R}}}}}$ are exponentially small. (If we ignore boundary effects, then the fundamental length scale is given by $\lambda$.) This allows us to write

$$\begin{aligned} G({\textbf{x}},{\textbf{x}}')&\sim \frac{1}{2\pi D}K_0\left( |{\textbf{x}}- {\textbf{x}}'|/\lambda \right) , \ {\textbf{x}}\ne {\textbf{x}}', \\ {\widehat{{{\mathcal {R}}}}}({\textbf{x}}',{\textbf{x}}')&\sim \frac{1}{2\pi D}\left( \ln \lambda + \ln 2 - \gamma _c\right) . \end{aligned}$$

Substituting these approximations into Eq. (4.6) implies that

4.9 $$\begin{aligned} A_j(\nu )&\sim \frac{\nu c_0}{1+\nu \left( \ln 2-\ln \rho _j/\lambda - \gamma _c\right) }\sum _{k=1}^N[{\varvec{K}}(\nu )^{-1}]_{jk}, \end{aligned}$$

where

4.10 $$\begin{aligned}{}[{\varvec{K}}(\nu )]_{jk}=\delta _{j,k}+ \frac{\nu K_0(|{\textbf{x}}_j-{\textbf{x}}_k|/\lambda )[1-\delta _{j,k}]}{1+\nu \left( \ln 2-\ln \rho _j/\lambda - \gamma _c\right) }. \end{aligned}$$

The asymptotic analysis has yielded an inner solution around each cluster that is expressed in terms of the unknown radii $\rho _j$, $j=1,\ldots ,N$. The latter are now determined self-consistently by imposing the conditions (2.5), which in 2D reduces to the implicit equations

4.11 $$\begin{aligned} 2\pi \rho _j D \frac{ A_j(\nu )}{\rho _j}= \epsilon ^2 \kappa _0 {u}_0 \pi \rho _j^2,\quad j=1,\ldots ,N. \end{aligned}$$

Substituting for $A_j(\nu )$ using Eq. (4.9) and noting that $u_0=2c_0/\epsilon ^2$ yields

4.12 $$\begin{aligned} \left( \frac{\rho _j}{\lambda }\right) ^2 \sim \frac{\nu }{1\!+\!\nu \left( \ln 2\!-\!\ln \rho _j/\lambda \!-\! \gamma _c\right) }\sum _{k=1}^N[{\varvec{K}}(\nu )^{-1}]_{jk}.\nonumber \\ \end{aligned}$$

The first factor on the right-hand side of Eq. (4.12) recovers the mean field Eq. (2.17). The second factor is the correction to mean field theory that involves diffusion-mediated interactions between the anchor points.

**Fig. 6:**
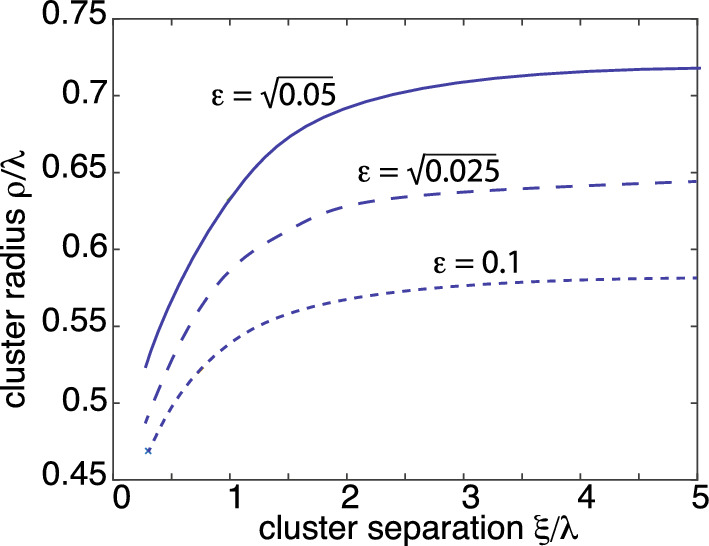
Pair of clusters of radius $\rho$ given by the solution to the non-perturbative Eq. (4.15). The radius $\rho$ is plotted as a function of cluster separation $\xi$ for different values of $\epsilon$ with $\epsilon ^2 =2c_0/u_0$. In the limit $\xi \rightarrow \infty$ we recover the mean-field results

As a simple illustration consider a pair of clusters with spatial separation $\xi =|{\textbf{x}}_1-{\textbf{x}}_2|$. If we ignore the effects of the exterior boundary, then both clusters have the same radius $\rho _1=\rho _2=\rho$. The matrix ${\varvec{K}}$ and its inverse are then simply

4.13a $$\begin{aligned} {\varvec{K}}(\nu )&=\left( \begin{array}{cc} 1 &{} \Gamma (\nu ,\xi )\\ \Gamma (\nu ,\xi ) &{}1 \end{array} \right) , \end{aligned}$$

4.13b $$\begin{aligned} {\varvec{K}}(\nu )^{-1}&=\frac{1}{1-\Gamma (\nu ,\xi )^2}\left( \begin{array}{cc} 1 &{} -\Gamma (\nu ,\xi )\\ -\Gamma (\nu ,\xi ) &{}1 \end{array} \right) , \end{aligned}$$

where

4.14 $$\begin{aligned} \Gamma (\nu ,\xi )= \frac{\nu K_0(\xi /\lambda )}{1+\nu \left( \ln 2-\ln \rho /\lambda - \gamma _c\right) }. \end{aligned}$$

It follows that $\rho$ is the solution to the implicit equation

4.15 $$\begin{aligned} \left( \frac{\rho }{\lambda }\right) ^2&\sim \frac{\nu }{1+\nu \left( \ln 2-\ln \rho /\lambda - \gamma _c\right) }\frac{1}{1+\Gamma (\nu ,\xi )}. \end{aligned}$$

In Fig. 6, we plot the numerical solution of Eq. (4.15) as a function of the cluster separation $\xi$ for $\epsilon ^2=0.01$, $\epsilon ^2=0.025$ and $\epsilon ^2=0.05$. The mean-field solution is recovered in the limit $\xi \rightarrow \infty$. It can be seen that the non-perturbative corrections to mean-field theory are significant.

## Asymptotic analysis of the 3D clustering model

Following the mean field analysis of the 3D model in Sect. 3.2, we introduce the rescalings $I_0={\overline{I}}_0/\epsilon$ and $c_0={\overline{c}}_0/\epsilon$ with ${\overline{c}}_0={\overline{I}}_0/\kappa _0$. Again, we use the version of the clustering model given by Eq. (3.2). It follows that Eq. (3.1a) becomes ${\varvec{\nabla }}^2 c-\kappa _0c=0$ while the boundary condition (3.1b) still holds with $c_j=-{\overline{c}}_0/\epsilon$ for all *j*. The outer solution is now expanded as

5.1 $$\begin{aligned} c({\textbf{x}})\sim c_1({\textbf{x}})+\epsilon c_2({\textbf{x}})+\ldots \end{aligned}$$

with

5.2a $$\begin{aligned} D{\varvec{\nabla }}^2 c_{\ell }({\textbf{x}})-\kappa _0c_{\ell }({\textbf{x}}) =&0,\quad {\textbf{x}}\in \Omega \backslash \{{\textbf{x}}_1,\ldots ,{\textbf{x}}_N\}, \end{aligned}$$

5.2b $$\begin{aligned} {\varvec{\nabla }}c_{\ell }({\textbf{x}})\cdot {\textbf{n}}&=0,\quad {\textbf{x}}\in \partial \Omega , \end{aligned}$$

together with singularity conditions as ${\textbf{x}}\rightarrow {\textbf{x}}_j$, $j=1,\ldots ,N$. In the inner region around the *j*-th cluster, we again introduce the stretched coordinates $\textbf{y}=\epsilon ^{-1}({\textbf{x}}-{\textbf{x}}_j)$ and set $C_j(\textbf{y}) =c({\textbf{x}}_j+\epsilon {\textbf{y}})$. Expanding the inner solution as

5.3 $$\begin{aligned} C_j({\textbf{y}}) =\frac{C_{j,0}({\textbf{y}})}{\epsilon }+C_{j,1}({\textbf{y}})+\ldots , \end{aligned}$$

we find that

5.4a $$\begin{aligned} {\varvec{\nabla }}_\textbf{y}^2 C_{j,\ell }&=0, \ \ell =0,1, \end{aligned}$$

5.4b $$\begin{aligned} {\varvec{\nabla }}_\textbf{y}^2 C_{j,\ell }&=\kappa _0 C_{j,\ell -2},\ \ell \ge 2,\quad {\textbf{y}}\in {{\mathbb {R}}}^3\backslash {{\mathcal {U}}}_j \end{aligned}$$

5.4c $$\begin{aligned} C_{j,\ell }({\textbf{y}})&=-{\overline{c}}_0\delta _{\ell ,0},\quad {\textbf{y}}\in \partial {{\mathcal {U}}}_j. \end{aligned}$$

Finally, the matching condition is that the near-field behavior of the outer solution as ${\textbf{x}}\rightarrow {\textbf{x}}_j$ should agree with the far-field behavior of the inner solution as $|{\textbf{y}}|\rightarrow \infty$, which is expressed as

$$\begin{aligned} c_1+\epsilon c_2 \sim \frac{C_{j,0}}{\epsilon }+ C_{j,1}+\ldots \end{aligned}$$

The iterative matching of the inner solution for $\ell =0,1$ with the outer solution proceeds along identical lines to Sect. 3.2 with the Green’s function $G_0({\textbf{x}},{\textbf{y}})$ replaced by $G({\textbf{x}},{\textbf{y}})$. Hence, we only summarize the results. The outer solution takes the form

5.5 $$\begin{aligned} c({\textbf{x}})\sim & {} - {4\pi } {\overline{c}}_0 D \sum _{k=1}^N\rho _k(1-\epsilon \chi _k)G({\textbf{x}},{\textbf{x}}_k), \end{aligned}$$

while the inner solution around the *j*-th cluster is

5.6 $$\begin{aligned} C_j(\rho )\sim {\overline{c}}_0 \bigg [\frac{1}{\epsilon } - \chi _j+O(\epsilon )\bigg ]\left( 1-\frac{\rho _j}{\rho }\right) . \end{aligned}$$

The final step is to determine the unknown radii $\rho _j$, $j=1,\ldots ,N$, by imposing the conditions (2.5), which in 3D reduces to the implicit equation

5.7 $$\begin{aligned} 4\pi \epsilon \rho _j^2 D {\overline{c}}_0 \bigg [\frac{1}{\epsilon } - \chi _j+O(\epsilon ) \bigg ]\frac{1}{\rho _j} = \epsilon ^3 \kappa _0 {u}_0 \frac{4\pi \rho _j^3}{3 }.\nonumber \\ \end{aligned}$$

Setting $u_0={\overline{u}}_0/\epsilon ^3$ then yields

5.8 $$\begin{aligned} \rho _j^2&=\frac{3 \lambda ^2{\overline{c}}_0}{{\overline{u}}_0}\bigg [1-\epsilon \chi _j+O(\epsilon ^2)\bigg ]. \end{aligned}$$

The *O*(1) contribution recovers the mean field result (2.23) whereas the $O(\epsilon )$ coefficient $\chi _j$, see Eq. (3.31), is the correction to mean field theory that involves diffusion-mediated interactions between the clusters. It follows that keeping the $O(\epsilon )$ term results in an implicit equation for the radii, which can also be solved perturbatively. Introducing the series expansion $\rho _j=\rho _j^{(0)}+\epsilon \rho _j^{(1)}+O(\epsilon ^2)$, we have

5.9 $$\begin{aligned} \rho _j^{(0)}=\rho _0\equiv \sqrt{\frac{3 \lambda ^2{\overline{c}}_0}{{\overline{u}}_0}}, \end{aligned}$$

and

5.10 $$\begin{aligned} \rho _j^{(1)} = - 2\pi \rho _0^2D \bigg ( R({\textbf{x}}_j,{\textbf{x}}_j)+ \sum _{k\ne j}^NG({\textbf{x}}_j,{\textbf{x}}_k)\bigg ).\nonumber \\ \end{aligned}$$

**Fig. 7:**
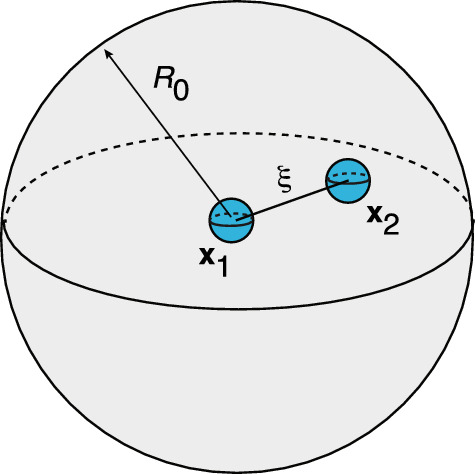
Pair of spherical clusters in a sphere of radius $R_0$. The first sphere is at the origin and the other is at a radial distance $\xi$ from the center

**Fig. 8:**
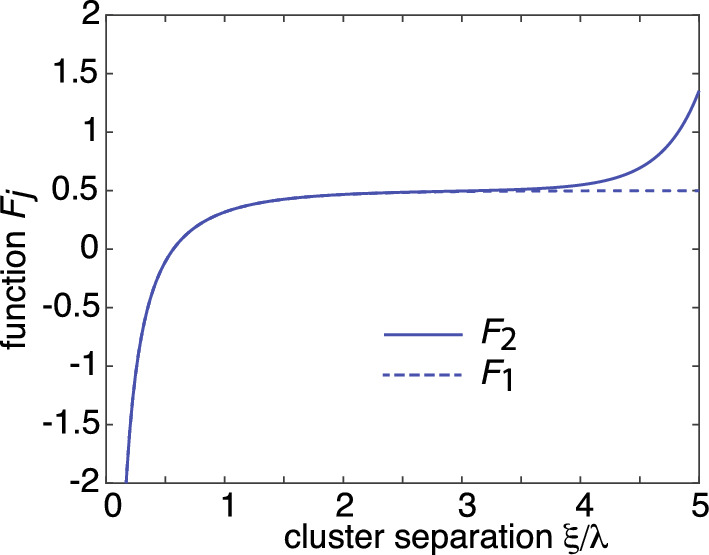
Plots of the multiplicative factors $F_j(\xi )$ defined in Eq. (5.18) as a function of radial separation $\xi$ for the configuration shown in Fig. 7. The radius of the spherical domain $\Omega$ is $R_0=5\lambda$

As a simple example, consider the 3D configuration shown in Fig. 7. The domain $\Omega$ is taken to be a sphere of radius $R_0$ with one cluster at the origin, ${\textbf{x}}_1={\varvec{0}}:=(0,0,0)$ and the other at ${\textbf{x}}_2={\varvec{\xi }}:= (\xi ,0,0)$ with $0< \xi <R_0$. The 3D Neumann Green’s function for the modified Helmholtz equation in the sphere is known explicitly [59]:

5.11 $$\begin{aligned} {G} ({\textbf{x}},{\textbf{x}}')&=\frac{{\mathrm e}^{-|{\textbf{x}}-{\textbf{x}}'|/\lambda }}{4\pi D|{\textbf{x}}-{\textbf{x}}'|}-{G}_\textrm{sp}({\textbf{x}},{\textbf{x}}'), \end{aligned}$$

with

5.12 $$\begin{aligned} {G}_\textrm{sp}({\textbf{x}}, {\textbf{x}}')&=\frac{1}{4\pi D \lambda }\sum _{n=0}^{\infty } (2n+1)P_n(\cos \theta )\frac{k_n'(R_0/\lambda )}{i_n'(R_0/\lambda )}\nonumber \\&\qquad \times i_n(|{\textbf{x}}|/\lambda ) i_n(|{\textbf{x}}'|/\lambda ). \end{aligned}$$

Here $P_n$ is a Legendre polynomial, ${\textbf{x}}\cdot {\textbf{x}}' =|{\textbf{x}}||{\textbf{x}}'|\cos \theta$, and $i_n,k_n$ are modified spherical Bessel functions,

5.13 $$\begin{aligned} i_n(x)=\sqrt{\frac{\pi }{2x}}I_{n+1/2}(x),\quad k_n(x)=\sqrt{\frac{2}{\pi x}}K_{n+1/2}(x).\nonumber \\ \end{aligned}$$

The regular part of the Green’s function is

5.14 $$\begin{aligned} R({\textbf{x}},{\textbf{x}})=-\frac{1}{4\pi D \lambda }-G_\textrm{sp}({\textbf{x}}, {\textbf{x}}). \end{aligned}$$

Using the identities

5.15 $$\begin{aligned} i_0(x)=\frac{\sinh x}{x},\quad k_0(x)=\frac{{\mathrm e}^{-x}}{x}, \quad i_n(0)=0, \ n>0,\nonumber \\ \end{aligned}$$

we see that

5.16 $$\begin{aligned} G({\varvec{0}},{\varvec{\xi }})&=\frac{1}{4\pi D \lambda }\left[ \frac{e^{-\xi /\lambda }}{ \xi /\lambda }- \frac{k_0'(R_0/\lambda )}{i_0'(R_0/\lambda )}i_0(\xi /\lambda )\right] . \end{aligned}$$

Further useful identities are

$$\begin{aligned} (2n+1) i'_n&=ni_{n-1}+(n+1)i_{n+1},\\ -(2n+1) k'_n&=nk_{n-1}+(n+1)k_{n+1}. \end{aligned}$$

Equations (5.9) and (5.10) imply that

5.17a $$\begin{aligned} \rho _1&=\rho _0+ 2\pi \epsilon D \rho _0^2\bigg ( \frac{1}{4\pi D \lambda }+G_\textrm{sp}({\varvec{0}}, {\varvec{0}})- G({\varvec{0}},{\varvec{\xi }})\bigg ), \end{aligned}$$

5.17b $$\begin{aligned} \rho _2&=\rho _0+ 2\pi \epsilon D \rho _0^2\bigg ( \frac{1}{4\pi D \lambda }+G_\textrm{sp}({\varvec{\xi }}, {\varvec{\xi }})- G({\varvec{0}},{\varvec{\xi }})\bigg ). \end{aligned}$$

It is convenient to rewrite these equations in the form

5.18 $$\begin{aligned} \frac{\rho _j-\rho _0}{\rho _0}=\epsilon \frac{\rho _0}{\lambda } F_j(\xi /\lambda ), \ j=1,2. \end{aligned}$$

The functions $F_j(\xi )$ are plotted in Figs.  8 for $R_0=5\lambda$. A number of observations can be made. (i) When the clusters are well separated, the radius of the first cluster approaches the mean-field value (2.23) since $F_1(\xi )\approx 1/2$. (ii) Away from the boundary, $\xi < R_0$, the two clusters have approximately the same radius, which is less than the mean-field value since $F_j(\xi ) < 1/2$. However, as the second cluster approaches the boundary, its radius exceeds the mean-field value, that is, $F_2(\xi )>1/2$. (iii) For sufficiently small $\xi$, we have $J_j(\xi ) < 0$ so that $\rho _j < \rho _0$.

## Discussion

In this paper, we used the theory of diffusion in singularly perturbed domains to calculate corrections to the mean field theory of 2D and 3D models of particle clustering in the presence of anchoring sites. We used matched asymptotics and Green’s function methods to derive implicit equations for the stationary cluster sizes given by Eqs. (4.12) and (5.8), respectively. The main result is that diffusion-mediated interactions between the clusters generate corrections to the mean-field average radius that depend on the positions of all the anchoring sites and the exterior domain boundary $\partial \Omega$. We illustrated the theory in the simple case of a pair of clusters, where we explored how the radii depended on cluster separation. We showed that corrections to mean field theory can be significant, particularly in 2D.

In future work, it would be interesting to consider configurations involving a large number of randomly distributed anchoring points. The numerical solutions for the radii could then be compared with detailed particle-based simulations in order to determine to what extent mean field corrections contribute to the distribution of cluster sizes. In Ref. [25], the distribution of cluster sizes was investigated by considering stationary solutions of a set of non-spatial Smoluchowski coagulation equations describing the aggregation of diffusing particles outside of the anchored domains. Such effects were incorporated into the mean-field model by introducing a typical radius $R_\textrm{typ}$ and a typical diffusivity $D_\textrm{typ}$ of diffusing clusters. Equations (2.10) and (2.11) were modified accordingly [25]:

6.1 $$\begin{aligned} D_\textrm{typ}{\varvec{\nabla }}^2 c(r)-\kappa _0 c(r)+I_0-\phi _0 J(R^*)&=0 \end{aligned}$$

for $r>R^*$, where $R^*=R+R_\textrm{typ}$ is the effective radius at which diffusing and anchored clusters fuse, $c(R^*)=0$, and (in the 2D case)

6.2 $$\begin{aligned} J(R^*):=2\pi R^* D_\textrm{typ}\partial _rc(R^*)=4\pi R^2 \kappa _0\phi _0. \end{aligned}$$

These modifications yielded mean field estimates for the average cluster size that were more consistent with the distribution of cluster sizes obtained using the rate equations. However, ideally one would like to account for the cluster size distribution using a fully spatial model. The asymptotic methods used in this paper provide a framework for achieving this. For example, one can incorporate $D_\textrm{typ}$ and $R_\textrm{typ}$ into the analysis of Sects. 4 and 5 by taking $D\rightarrow D_\textrm{typ}$, $\lambda \rightarrow \sqrt{D_\textrm{typ}/\kappa _0}$, $R_j^*=\epsilon \rho _j$ and $R_j=\epsilon (\rho _j-\rho _\textrm{typ})$. A more difficult challenge is extending the theory to a fully spatial Smoluchowski coagulation model with anchoring points.

One natural generalization of the model given by Eq. (2.2) would be to modify the absorbing boundary condition on the surface of each cluster. For example, we could replace the Dirichlet boundary condition $c({\textbf{x}})=0$ by the Robin boundary condition $D{\varvec{\nabla }}c({\textbf{x}})\cdot {\textbf{n}}_j=\kappa _0 c({\textbf{x}})$ for ${\textbf{x}}\in \partial {{\mathcal {U}}}_j$, where $\kappa _0$ is the surface reactivity. (The analysis of diffusion in domains with partially absorbing traps has been analyzed elsewhere within the context of narrow capture problems [60].)

As originally shown by Ward and Keller [45] within the context of 2D and 3D eigenvalue problems, it is possible to generalize the asymptotic analysis presented in this paper to more general cluster shapes such as ellipsoids by applying classical results from electrostatics. For example, given a general shape ${{\mathcal {U}}}_j \subset {{\mathbb {R}}}^3$, the solution $w({\textbf{y}})$ to Eq. (3.25) has the far-field behavior

6.3 $$\begin{aligned} w({\textbf{y}})\sim \frac{{{\mathcal {C}}}_j}{|{\textbf{y}}|}+\frac{\textbf{P}_j\cdot {\textbf{y}}}{|{\textbf{y}}|^3}+\ldots \text{ as } |{\textbf{y}}|\rightarrow \infty . \end{aligned}$$

Here ${{\mathcal {C}}}_j$ is the capacitance and $\textbf{P}_j$ the dipole vector of an equivalent charged conductor with the shape ${{\mathcal {U}}}_j$. (For a sphere, ${{\mathcal {C}}}_j=\rho _j$ and $\textbf{P}_j=0$.) It turns out that the $O(\epsilon )$ and $O(\epsilon ^2)$ contributions to the inner solution only depend on ${{\mathcal {C}}}_j$ so that the various results of Sect. 3.2 still hold on making the replacements $\rho _j\rightarrow {{\mathcal {C}}}_j$ for $j=1,\ldots ,N$. Similarly, in 2D one can deal with non-circular shapes by setting $\nu _j=-1/\ln \epsilon d_j$ with $d_j$ the associated logarithmic capacitance.

An implicit assumption of our analysis was that the non-equilibrium steady-state solution consisting of multiple clusters is dynamically stable. This is consistent with detailed particle-based simulations of cluster formation in the presence of anchoring sites provide strong evidence that multi-cluster states are stable and that analytical approaches capture how cluster sizes depend on the density of anchoring sites, for example, [25]. Moreover, it should be possible to establish stability by extending the analysis of a singularly perturbed diffusion problem that arises in a model of quorum sensing [58].

Finally, another interesting open problem is to consider the transient formation of clusters and time-dependent processes that occur by possibly solving the full time-dependent model (2.2), rather than the time-independent version. So far the techniques of diffusion with strongly localized perturbations have mainly been applied to steady-state problems, with a few exceptions in the narrow capture literature where one typically works in Laplace space. The examples of protein clustering and Ostwald ripening suggest that it would be valuable to develop asymptotic methods where dynamical effects are important to understand.
